# Allogeneic whole-eye transplantation: advancements, challenges, and future directions in vision restoration

**DOI:** 10.3389/fmed.2025.1691259

**Published:** 2025-11-12

**Authors:** Lingxi Wei, Wenqi Yan, Kai Zhang, Fei Gao, Zhuoling Li, Ruonan Pan, Zhengwei Zhang, Xiaogang Wang

**Affiliations:** 1 Shanxi Medical University, Taiyuan, Shanxi, China; 2 Shandong University, Jinan, Shandong, China; 3 Department of Ophthalmology, Wuxi Clinical College, Nantong University, Wuxi, China; 4 Department of Ophthalmology, Jiangnan University Medical Center, Wuxi, China; 5 Department of Cataract, Shanxi Eye Hospital, Shanxi Medical University, Taiyuan, China

**Keywords:** allogeneic, whole-eye transplantation, vision loss, progress, challenges

## Abstract

Vision loss remains a significant global health burden, primarily driven by irreversible ocular conditions such as age-related macular degeneration (AMD), glaucoma, severe ocular trauma, and intraocular malignancies. Despite advances in retinal prosthetics and stem cell-based therapies, current treatment options are still limited in their ability to fully restore visual function. Allogeneic whole-eye transplantation (WET) has recently gained attention as a novel and potentially transformative strategy for vision restoration. This review synthesizes recent progress in the field, including advancements in microsurgical techniques, immunosuppressive protocols, and neural integration strategies, drawing on evidence from both preclinical animal models and emerging human studies. Key components, including optic nerve (ON) regeneration, vascular anastomosis, immune tolerance, and donor–recipient matching, are critically examined. Furthermore, we address ongoing barriers, including graft viability, chronic rejection, central visual pathway rewiring, and ethical considerations surrounding the procurement of donor eyes. While substantial milestones have been achieved, particularly in experimental settings, clinical translation remains in its early stages. This review highlights current limitations and proposes future directions for multidisciplinary research aimed at overcoming these challenges and advancing WET toward clinical reality.

## 1 Introduction

Visual perception and normal visual function play a crucial role in daily functioning and overall quality of life (1). Irreversible ocular conditions such as age-related macular degeneration (AMD), glaucoma, severe ocular trauma, and intraocular malignancies are leading causes of permanent vision loss worldwide (2). According to the World Health Organization’s 2019 report, more than 2.2 billion people worldwide have visual impairment or blindness, placing a significant burden on healthcare systems and societies (3). Conventional treatments, including pharmacotherapy, laser photocoagulation, and surgical interventions, can slow disease progression but often fail to restore vision once retinal or optic nerve damage has occurred. Meanwhile, emerging technologies such as retinal prostheses and stem cell therapies have shown promise in experimental models but are limited in their clinical efficacy, especially in restoring complex visual perception (4).

Allogeneic whole-eye transplantation (WET) has gained increasing scientific and clinical interest as a radical yet potentially transformative solution for irreversible vision loss. Involving the complete replacement of the ocular globe, WET endeavors to preserve facial esthetics while concurrently aiming to restore visual function through the meticulous reconnection of the donor eye’s neurovascular structures with the recipient’s central nervous system. A pivotal achievement in this domain occurred on 9 November 2023, when the team led by Eduardo Rodriguez at NYU Langone Health performed the world’s first WET in a human subject. Although complete visual restoration was not achieved, the patient exhibited partial sensory responsiveness, indicating a promising advancement in this innovative surgical field (5, 6).

Despite its potential, WET faces substantial challenges, including optic nerve regeneration, immune rejection, re-establishment of retinal perfusion, donor–recipient tissue compatibility, and ethical considerations surrounding organ donation. This review presents a comprehensive analysis of the current advancements in WET, emphasizing progress in preclinical animal models, emerging clinical reports, and innovations in microsurgery, immunosuppression, and neuroregeneration. It also delineates the critical challenges that must be addressed for WET to become a viable clinical option. Furthermore, it investigates prospective research avenues essential for translating this vision-restoring strategy into widespread clinical practice.

## 2 Review of previous studies

The concept of WET has evolved significantly over the past several decades, transitioning from basic experimental models to complex preclinical and clinical applications. Initial investigations into ocular regeneration were largely conducted in cold-blooded vertebrates, such as newts and frogs, which exhibit extraordinary regenerative capacity in ocular tissues, particularly the retina and optic nerve (7). These models provided foundational insights into the cellular mechanisms of eye regrowth but were limited in translational value due to significant anatomical and immunological differences from humans. To address these limitations, researchers turned to mammalian models, which offer a more physiologically relevant framework for studying immune responses, neurovascular integration, and donor–recipient compatibility. In a landmark study, a composite eye-orbit transplantation model in rodents was developed, incorporating the globe, optic nerve, eyelids, and associated vasculature into a single graft (8). This comprehensive model enabled a detailed investigation into vascular anastomosis, immune tolerance, and neural regeneration following WET, laying the groundwork for future translational efforts in humans. Subsequent studies have refined surgical techniques and improved post-transplant management, including immunosuppressive protocols and neurotrophic factor delivery, to enhance graft survival and promote functional recovery. Notably, advances in microsurgical precision, stem cell therapy, and neuroengineering have further propelled the feasibility of WET as a vision-restorative intervention. The first human WET study demonstrated the partial preservation of sensory function by using adult stem cells to aid in optic nerve junction repair (6). While full visual recovery remains elusive, this procedure has validated the potential of earlier preclinical findings and has reignited scientific interest in the clinical translation of WET (5).

### 2.1 Early animal experiments

Pioneering studies on WET have long relied on cold-blooded vertebrates, particularly amphibians such as *Rana pipiens*, *Amblystoma*, and *Xenopus*, due to their extraordinary regenerative abilities and experimental tractability. These species offer distinct advantages for ocular regeneration research, including high neural plasticity, ease of manipulation, and ethical and economic feasibility compared to higher vertebrates.

Early in the 20th century, Keeler (1924–1928) performed over 50 eye transplantation surgeries on *Rana pipiens* (leopard frogs), although none resulted in functional vision recovery (9). Progress was made by Roger Sperry, who demonstrated partial visual recovery in amphibians, achieving restored visual responses in 2 of 30 tadpoles and 12 of 21 adult tailless newts following WET procedures (10, 11). These results provided early evidence of neural reconnection between transplanted eyes and the host brain. Further advancements were made by Stone (12–14), who explored cross-species transplantation between *Amblystoma punctatum* (the spotted salamander) and *Ambystoma tigrinum* (the tiger salamander). His findings revealed that the donor eyes of tiger salamanders grew faster and exhibited pupillary light reflexes after metamorphosis. In one of the most promising early outcomes, visual function was restored in 31 of 35 transplanted salamanders (12, 14). Subsequent work demonstrated functional vision in eight *Triturus* hosts with donor eyes from Ambystoma (15). The 1980s saw further refinements. Harris (16) used mutant salamanders to demonstrate regrowth of optic pathways, showing varied projection patterns, including ipsilateral, contralateral, and bilateral wiring, thus illustrating the remarkable adaptability of the amphibian visual system. Pietsch (17) also made a significant contribution, demonstrating that eye transplantation in spotted salamander larvae led to the restoration of visual function in 18 of 22 recipients. In the early 2000s, Sedohara et al. (18) induced eye formation in Xenopus embryos *in vitro* and transplanted these to tadpoles. Three of fifteen embryos retained their eyes through metamorphosis and regained vision (18). Building on this, Blackiston et al. (19) successfully generated ectopic eyes along the body axis of *Xenopus laevis* tadpoles. Astonishingly, 95% of these cases demonstrated visual function despite the eyes being positioned outside the cranial region, indicating that the central nervous system can interpret input from non-traditional locations (19). Collectively, these foundational experiments highlight the capacity for neural plasticity and functional integration in regenerative species. They have laid essential groundwork for transitioning from amphibian to mammalian models in the pursuit of clinically viable WET (Table 1).

**Table 1:** Important historical experiments of whole-eye transplantation in cold-blooded animals.

Date	Study	Animal models	Animal numbers	Transplantation sites	Transplantation methods	Methods of visual acuity assessment	Visual recovery status
1929	Keeler C. (9)	Frog	54	Right eye sockets	Reimplanted	Action-current response	0 of 54(0%) frogs restored normal vision
1930	Stone LS. (12)	Amblystoma	41	Left/right orbital cavity	Allogeneic transplantation	Restoration of visual acuity in behavioral responses	6 of 6 (100%) were with recovered visual function
1937	Stone LS., et al. (13)	Salamander larvae	63	Bilateral eye sockets	Transplants and reimplantation	Vision restoration testing	Transplanted: 25 of 26 (96.2%) and reimplanted:7 of 9 (77.8%) with recovered visual function
1938	Stone LS. (20)	Salamander	More than 350	Bilateral eye sockets	Enucleation of the eyeball and reimplantation	Constriction reflex of pupils in response to light stimulus	NA
1940	Stone LS., et al. (21)	*Amblystoma punctatum*	92	Left/right orbital cavity	Replantation and transplantation	Constriction reflex of pupils in response to light stimulus	Reimplanted: 33 of 33 (100%) and transplanted: 54 of 59(91.5%) recovered visual function
1942	Weiss P (22)	*California newt*	45	Left/right orbital cavity	Excision and transplantation	Stress response (eyelid closure)	40 of 45 (88.9%) *California newts* with recovered visual function
1943	Stone LS. et al. (23)	Salamander	104	Bilateral eye sockets	Total eye excision and reimplantation	Constriction reflex of pupils in response to light stimulus	Transplanted: 11 of 26 (42.3%) and reimplanted: 24 of 76 (31.6%) unrecovered visual function
1945	Stone LS. et al. (14)	Tadpoles and adult animals of tailless salamanders	34 Amblystoma hosts with Triturus eyes and 46 Triturus hosts with Amblystoma eyes	Left/right orbital cavity	Homologous allogeneic transplantation	Behavioral response test	8 of 80 (10.0%) salamanders recovered visual function
1945	Sperry, RW. (11)	Tadpoles and adult animals of the tailless newts	30tadpoles + 21adult tailless salamanders	Left/right orbital cavity	Autologous contralateral transplantation	Leap onto lures after metamorphosis	38 of 42 (90.5%)with recovered visual function in tailless newts
1946	Stone LS. (24)	Salamander	35	Right orbital cavity	Enucleation and reimplanted in the correct orientation	Constriction reflex of pupils in response to light stimulus	NA
1949	Sperry RW. (25)	Fish	22	Bilateral eye sockets	Total eye excision	Object recognition (optical motion tracking)	20 of 22 (100.0%) eyes were with recovered visual function
1956	Bytinski-Salz H. (26)	Brook lamprey	30	Bilateral eye sockets	NA	NA	NA
1957	Heath HD. (27)	Salamander	14	Bilateral eye sockets	Organ exchange between embryos and larvae	NA	NA
1964 (July)	Stone LS. (28)	Salamander	11	Left/right orbital cavity	Enucleation of the reciprocal host, compare the transplanted eye with the normal donors left eye	Constriction reflex of pupils in response to light stimulus	8 of 11 (72.8%) salamanders recovered visual function
1964 (December)	Stone LS. (29)	Salamander	26	Bilateral eye sockets	Enucleation of the reciprocal host, compare the transplanted eye with the normal donor’s left eye	Constriction reflex of pupils in response to light stimulus	10 of 26 (28.5%) salamanders recovered visual function
1968	Schneider CW, et al. (30)	Salamander	12	Left/right orbital cavity	Enucleation of one eye post-harvest and transplantation	Constriction reflex of pupils in response to light stimulus	12 of 14 (85.7%) salamanders recovered visual function
1982	Harris, W. A. (16)	Eyeless salamander	23	Left/Right orbital cavity	Allogeneic transplantation	Anatomical and physiological observations	NA
1988	Pietsch P, Schneider CW. (17)	Ambystoma larvae	66	Bilateral eye sockets	Removal of eyes from 22 spotted salamander larvae and subsequent eye transplantation	Restoring skin camouflage responses to environmental testing	46 of 66 (69.7%) eyes were with recovered visual function (heterograft)
2003	Sedohara, A., et al. (18)	*Xenopus*	15	The tadpole’s physiology	*In vitro* induction of eye transplantation and allogeneic transplantation	Brightness stimulation	Implanted: 3 of 15 (20.0%) eyes recovered visual function
2013	Blackiston, D. J., et al. (19)	*Xenopus*	NA	Body axis of *Xenopus*	Eye primordia grafts to create ectopic eyes along the body axis	Light-mediated learning assays with an automated machine vision and environmental control system	6 of 31 (19.35%) eyes exhibited light-mediated learning

NA = Not available.

#### 2.1.1 Pioneering work by Stone

The foundational work of Dr. Louis S. Stone in the early 20th century played a pivotal role in establishing the principles of ocular transplantation and optic nerve regeneration. His research on amphibian models, particularly salamanders, demonstrated the biological feasibility of eye graft survival and partial visual function restoration, making a substantial contribution to the early development of WET as a scientific concept.

In 1930, Stone performed interspecies eye transplants between *Amblystoma punctatum* (the spotted salamander) and *Amblystoma tigrinum* (the tiger salamander), observing that the transplanted eyes continued to grow in the host and regained key morphological features, such as pigmentation, corneal transparency, and retinal development. Notably, the transplanted eyes responded to light stimuli post-metamorphosis, suggesting functional neural integration (12). By 1937, Stone provided clear evidence of optic nerve regeneration, as transplanted eyes re-established connections with the host brain, enabling signal transmission necessary for basic visual responses (13). He further demonstrated in 1945–1946 that eyes enucleated and subsequently grafted into salamander hosts could elicit pupillary light reflexes, indicating the restoration of at least partial visual function (24). In a later set of experiments published in 1964, Stone carried out reciprocal eye transplantation procedures among various salamander species. He reported that 30.8% of transplanted eyes exhibited a constriction reflex to direct light stimulation—a quantifiable sign of recovered visual function (29). These findings were among the first to associate anatomical regrowth with measurable physiological responses, reinforcing the regenerative potential of the optic nerve in amphibians. Stone’s series of experiments laid essential groundwork for future research into optic nerve repair, neural plasticity, and the development of transplantation techniques. His work continues to influence current efforts in mammalian eye transplantation and regenerative ophthalmology, providing a historical foundation for modern advances in allogeneic WET.

#### 2.1.2 Evolution of mammalian models in WET research

While early WET research focused on amphibians due to their regenerative abilities, translating these findings into mammalian models has posed significant challenges. The history of WET in mammals dates back to 1886, when May conducted the first documented attempt to transplant eyes in rabbits. These initial efforts failed due to surgical complications, lack of vascular anastomosis, and absence of immunological understanding, resulting in no graft survival or visual recovery (31). In the ensuing decades, rodent models emerged as a primary research focus. Early attempts at WET in rats by Koppanyi and Baker and subsequently by Freed and Wyatt yielded limited retinal survival and no demonstrable restoration of visual function (32, 33). These persistent challenges were primarily ascribed to deficiencies in microsurgical techniques that effectively maintain the retinal vascular perfusion.

In 2009, Shi et al. achieved short-term retinal reperfusion and recorded transient electroretinographic (ERG) activity in swine models; however, long-term viability and functional vision were not restored (34). In 2012, Polat et al. reported successful transplantation of ocular tissue in rats, showing early signs of tissue recovery and retinal preservation (35). This was followed by the work of Zor et al. (36), who achieved 100% graft survival and persistent retinal responses for up to 30 days in Sprague–Dawley rats, indicating potential optic nerve regeneration and improved host-graft integration. To address the inherent size and anatomical constraints presented by rodent models, researchers subsequently shifted their focus to larger mammalian models, specifically sheep and pigs. In 2023, Sakarya et al. conducted WET in sheep, successfully maintaining retinal arterial perfusion. Despite this achievement, no functional visual recovery was observed, which indicated that vascular reconnection alone was insufficient to restore vision (37). These mammalian studies, summarized in Table 2, reflect the iterative progress in surgical technique, immunological management, and neurovascular integration that continue to move WET closer to clinical viability.

**Table 2:** Important historical experiments of complete eye transplantation in mammals.

Time	First author	Model organisms	Animal numbers	Transplantation sites	Transplantation methods	Vision testing	Visual recovery status
1886	May CH. (38)	Rabbit	24	Eye socket	NA	Object recognition	6/24 eyes (25%) survived, 0 of 24 regained vision
1924	Koppányi T, et al. (32)	Rat	25	Eye socket	Excision and transplantation	Corneal reflex	2/25 eyes (8%) recovered corneal reflex, 0 of 25 rats regained vision
1971	Burns RP, et al. (39)	Human (living human)	1	Eye socket	Autologous transplantation	NA	0/1 eye (0%) with recovered visual function
1980	Freed WJ, et al. (33)	Rat	12	Adult brains	Allogeneic transplantation (transplantation of whole eyes from fetal rats to blind adult brains)	electroretinograms (ERGs)	3/9 eyes (33.3%) with light-evoked potential 0 of 12 rats regained vision
1980	Sher H, et al. (40)	Rat, Canine	25	The femoral artery and vein of rats	Canine eyes anastomosed ectopically; Microsurgical techniques	Microscopic examination, fluorescein angiography	25/25 eyes (100%) with active circulation, 0 of 25 animals regained vision
1981	Sher H. (41)	Sheep	20	Eye socket	Autologous transplantation with vascular anastomosis	Microscopic examination, fluorescein angiography	40/40 eyes (100%) with active circulation; 5/20 fluorescein angiography showing continuous filling 0 of 20 animals regained vision
2009	Shi J, et al. (34)	Swine	20	Neck region	Vascular anastomosis following enucleation	Light reaction(ERG activity)and optic nerve responses	Electroretinogram and optic nerve responses were partially recovered 0 of 20 animals regained vision
2012	Polat, R.M., et al. (35)	Rat	12	Neck region	Homologous allogeneic transplantation	Macroscopy, MR, and histopathologic examination	Nerve coaptation sections showed severe degradation, with no evidence of regeneration. Eyeball loses its volume by 35% 0 of 12 animals regained vision
2016	Davidson EH, et al. (42)	Human (cadaver)	8	Eye socket	Measures edicle lengths and calibers	NA	The technical feasibility of cadaveric donor procurement and transplantation to cadaveric recipients was established.0 of 8 animals regained vision
2018	Siemionow M, et al. (43)	Human (cadaver)	5	Eye socket	Vascularized ectopic transplantation	Laser-assisted indocyanine green angiography	Confirmed the feasibility of composite eyeball-periorbital transplantation in the clinical setting.0 of 5 animals regained vision
2019	Zor F, et al. (36)	Mouse	5	The entire globe, adnexa, optic nerve(ON), and periorbital soft tissues	Vascularized ectopic transplantation	Angiography, MRI	All eyes (100%) survived 0 of 5 animals regained vision
2020	Bravo MG, et al. (44)	porcine cadaver	NA	NA	Extracorporeal transplantation, microvascular anastomosis, and nerve anastomosis	NA	NA
2022	Badaro E, et al. (45)	Rabbit	6	Eye socket	A novel technique to be used in rabbits	ERG activity	An ERG test with durations of 296 and 302 seconds.0 of 6 animals regained vision
2022	Komatsu C, et al. (46)	Rat	7	Eye socket	In vivo imaging model system, manganese-enhanced magnetic resonance imaging (MEMRI)	MRI/ion transport along the optic tract	Anterograde manganese transport after WET.0 of 7 animals regained vision
2023	Daniel J. Ceradini et al. (6)	Human	1	Eye socket and face	Personalized surgical devices; Vascularized composite allotransplantation; Globe transplantation	Reperfusion and viability of the whole eye and facial allografts, retinal function, and incidence of acute rejection.	The transplanted eye showed survival and retinal light response but resulted in no light perception.

#### 2.1.3 Target effectiveness difference between animal models and human experiments

Animal models are invaluable for verifying basic mechanisms and gathering essential technical parameters. Mammalian models, for instance, have successfully demonstrated the feasibility of retinal arterial perfusion and partial sensory recovery. This provides a crucial foundation for human surgeries concerning surgical techniques, immunological management, and neurovascular integration, thereby advancing the clinical translatability of WET. However, a significant “translational gap” persists between animal models and human clinical requirements. First, differences in physiological structures often lead to inadequate technical adaptability. Second, a mismatch in evaluation systems restricts the effective translation of clinical value. Animal models predominantly use “retinal structural survival” and the “presence of electrophysiological signals” as their primary evaluation metrics (34–36). In contrast, human WET necessitates the integration of dual goals of function and esthetics (43). This not only requires ensuring adequate blood supply to the graft and restoring periorbital sensation and eyelid movement function in patients but also demands facial appearance restoration, all of which extend beyond the current scope of animal model evaluation.

#### 2.1.4 Concept changing all the time

Artificial eye prostheses offer only static esthetic restoration, lacking periorbital sensation or visual function and often lead to tissue irritation (47). In contrast, WET technology represents a significant leap from mere structural replacement to functional repair. Emerging allogeneic WET transplantation shows promise in ensuring graft survival, achieving partial sensory recovery, and enhancing patients’ facial appearance and periorbital sensation post-surgery (6). Nevertheless, several challenges persist. The scarcity of suitable donor samples restricts technological advancement, while donor matching and managing organ ischemia time present significant ethical and resource constraints. Furthermore, the inherently low efficiency of human optic nerve regeneration and unresolved long-term immune rejection issues underscore the critical need for additional clinical cases to optimize treatment protocols and improve patient outcomes (48).

### 2.2 Technological advancements

#### 2.2.1 Evolution of surgical techniques

The evolution of surgical techniques in WET has been instrumental in advancing the feasibility of this complex procedure. Early foundational studies in the 1920s and 1930s by researchers such as Keeler, Stone, and Sperry demonstrated the potential for transplanted ocular tissue to survive and regain basic structural and functional features, particularly in cold-blooded animals (9, 10, 12). Significant progress was made in the mid-20th century through the pioneering work of Louis S. Stone and Roger W. Sperry. They introduced key innovations, including allogeneic transplantation models, photocoagulation techniques, and optic nerve reconnection protocols. These advancements led to improved graft survival and often resulted in near-complete recovery of light sensitivity and visual reflexes in cold-blooded species (10, 15, 24, 29). However, translating these techniques to mammalian models proved more complex. While rodent models have been crucial in refining blood supply reconnection, neural integration with the visual cortex remains a significant bottleneck (49). Contemporary WET research continues to focus on enhancing graft viability, mitigating ischemia–reperfusion injury, and stimulating neuroplasticity within the optic nerve and central visual pathways. Current investigations explore techniques such as neurotrophic factor delivery, stem cell support, and three-dimensional microvascular reconstruction to achieve more consistent and functional outcomes. These cumulative advancements in surgical precision and biological integration have established the groundwork for successful human WET procedures and will continue to guide the development of next-generation strategies aimed at restoring vision through complete ocular transplantation.

#### 2.2.2 Improvements in organ preservation methods

The success of WET is closely tied to advancements in organ preservation techniques, which reduce ischemic injury and improve post-transplant outcomes. The history of organ preservation dates back over two centuries to César Julien Jean Le Gallois, who proposed early theories on the viability of organs outside the body. However, modern preservation techniques were revolutionized in the 1960s with the introduction of static cold storage (SCS), which enabled hypothermic preservation, significantly extending the viability of donor organs by slowing down metabolic processes (50). More recently, dynamic machine perfusion (DMP), a technique that uses blood-based or acellular perfusates at near-physiological temperatures, has emerged as a superior alternative to SCS. DMP not only maintains organ viability but also facilitates real-time assessment and even repair of marginal grafts before transplantation, offering new hope for improving transplant success rates (51).

In ophthalmology, preservation methods have progressed in parallel with advancements in corneal and ocular surface transplantation. The amniotic membrane has become a widely used biological scaffold for ocular surface reconstruction. For penetrating keratoplasty, corneoscleral buttons are typically preserved using hypothermic storage (2–6 °C) for up to 7–10 days or organ culture at 31–37 °C for extended periods of up to 4 weeks. Both approaches have demonstrated comparable graft survival rates, although outcomes are influenced by factors such as donor tissue quality, geographic distribution, and local clinical practices (52). In the context of WET, recent research has focused on optimizing both graft procurement and preservation protocols. Anatomical studies have helped refine surgical flap designs to improve donor eye retrieval and vascular access. Rodent models have shown that heterotopic whole-eye allografts can survive for up to 30 days with preserved retinal architecture, while large animal models demonstrate that retinal ganglion cells (RGCs) can tolerate ischemia for up to 105 min at physiological temperatures, especially when cold storage techniques are applied (6, 53). Furthermore, cold preservation has been shown to enhance ischemic tolerance in bovine RGCs, supporting the development of new cold perfusion strategies tailored to ocular tissue. Emerging techniques now aim to preserve critical parameters such as retinal perfusion, intraocular pressure, and optic nerve integrity while minimizing immunogenicity and risk of graft rejection. These innovations are crucial for maintaining retinal function and light responsiveness after transplantation and are paving the way for the clinical implementation of successful WET procedures.

### 2.3 Key research achievements and milestones

**Figure 1:**
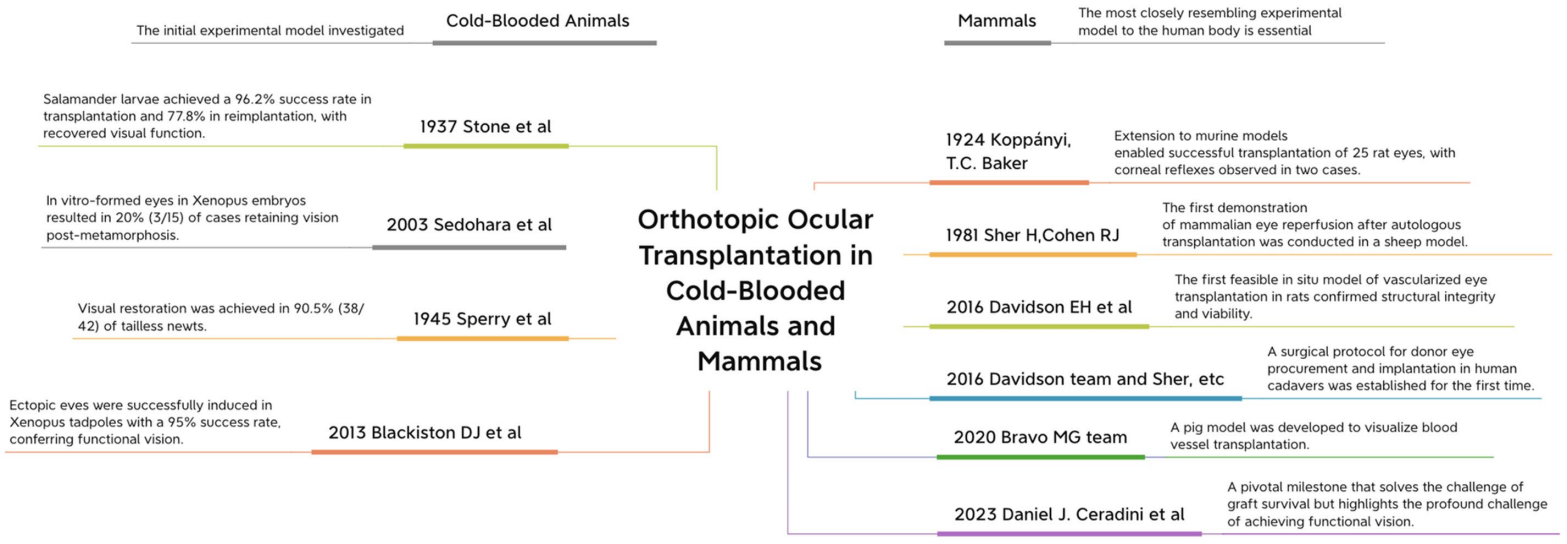
A historical review of WET in cold-blooded and mammalian animals: this review provides a timeline of key experiments and their significant implications. The left side represents cold-blooded animal studies, and the right side highlights advancements in mammalian research.

Research into WET transplantation has yielded substantial progress, especially in refining surgical methodologies and integrating biological elements. Figure 1 highlights critical achievements from this ongoing research, demonstrating the sequential advancements that have elevated WET to its current state. Despite these successes, the objective of restoring complete functional vision remains an area requiring further investigation.

## 3 The world’s first WET surgery in humans (Figure 2)

**Figure 2:**
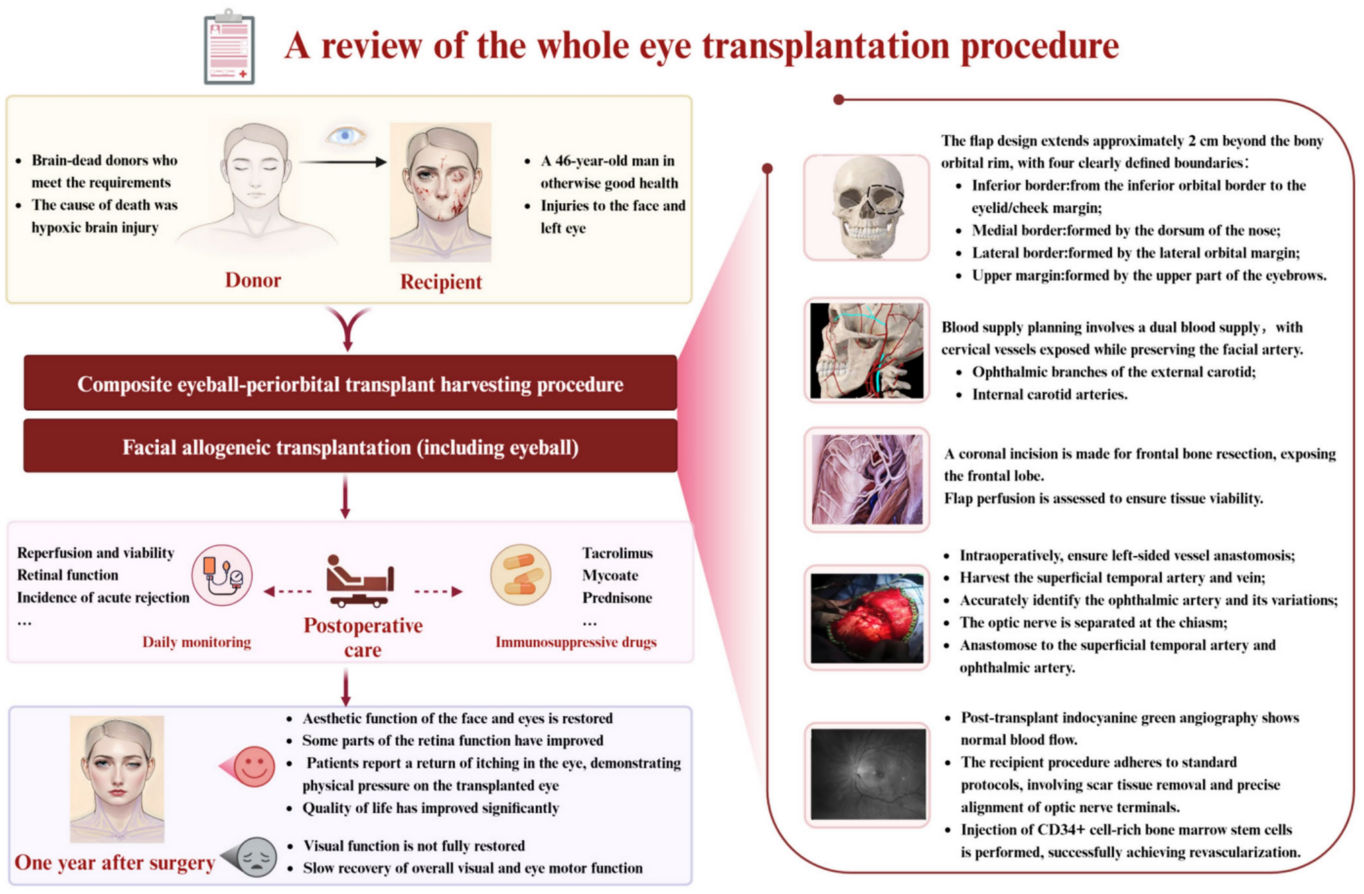
Review of allogeneic complete eye transplant surgery: allogeneic complete eye transplantation involves the harvesting and transplantation of composite eyeball-orbit tissues. This figure summarizes the preoperative preparations, surgical procedures, postoperative care methods, and the outcomes associated with the transplantation [29].

The pioneering WET on A. James, a patient who suffered severe facial and left eye trauma from high-voltage power lines in June 2021, represents a landmark in reconstructive surgery. In May 2023, NYU performed this intricate procedure following the identification of a suitable brain-dead donor (5, 6). Donor selection involved rigorous criteria, including the absence of neurological function, traumatic injury, and intracranial hemorrhage, alongside careful immunological matching to minimize rejection risks.

The surgical strategy for this composite eye-periorbital transplant integrated microsurgical, craniofacial, and neurovascular techniques. A key aspect involved a meticulous flap design, extending approximately 2 cm beyond the orbital rim, to ensure adequate margins and vascular perfusion via branches of both external and internal carotid arteries. Crucially, the procedure included frontal bone resection to facilitate direct access and precise coaptation of the optic nerve, along with intraoperative indocyanine green angiography for perfusion assessment. A significant innovation was the application of CD34^+^-enriched stem cells injected at the optic nerve junction, aimed at promoting axonal regeneration and mitigating ischemic damage. The comprehensive 21-h procedure, with a cold ischemia time of 2 h and 59 min, also incorporated immunosuppression with thymoglobulin, rituximab, tacrolimus, mycophenolate mofetil, and prednisone.

One year post-surgery, the patient demonstrated successful restoration of facial and ocular esthetic functions, with no acute rejection. While complete visual function was not achieved, normal intraocular pressure was maintained, and magnetic resonance imaging (MRI) and optical coherence tomography (OCT) confirmed stable eyeball volume. Electrophysiological tests (ERG and visual evoked potential (VEP)) indicated partial functional improvement in the visual pathway, suggesting potential neural integration (Figure 2). Despite persistent itching in the orbital region, the gradual recovery of eyelid and facial muscle function significantly improved the patient’s quality of life. This case highlights critical advancements in WET, particularly in achieving graft survival and laying the groundwork for future visual restoration efforts.

## 4 Discussion

### 4.1 Major challenges in WET

WET presents several significant challenges, particularly in nerve regeneration, immunological rejection, restoring retinal blood supply, and surgical complexity.

#### 4.1.1 Nerve regeneration and visual signal processing

Optic nerve regeneration remains one of the most formidable challenges in WET, primarily due to the intricate complexities involved in repairing both the central and peripheral nervous systems. Post-injury, RGC axons rapidly degenerate, a process exacerbated by their limited regenerative capacity, disrupted organelle dynamics, and inadequate neurotrophic support. These factors collectively culminate in irreversible RGC loss and profound vision impairment (54, 55). To counteract this, various strategies have been investigated to foster axonal regeneration and RGC survival, including gene therapy, neurotrophic factor administration, and stem cell-based interventions. Notably, combinatorial approaches leveraging stem cells and exosome-based therapies have demonstrated significant promise in enhancing neuronal survival and facilitating axonal growth (56).

Despite advancements in maintaining transplanted eye viability and inducing localized nerve regeneration, achieving functional reconnection between the regenerated optic nerve and the brain’s visual centers remains a critical and unresolved hurdle (57, 58). Successful optic nerve regeneration alone is insufficient; precise anatomical alignment and functional integration of the donor and recipient optic nerves are paramount for restoring vision. This necessitates not only anatomical reconnection but also the reestablishment of neural pathways capable of accurately transmitting visual information to the brain (59).

Furthermore, neuroplasticity and adaptation present additional significant obstacles. Following transplantation, the visual system frequently undergoes substantial reorganization, compelling the patient to “relearn” the interpretation of visual stimuli. Discrepancies between visual inputs from the transplanted eye and pre-injury perception, stemming from alterations in neural architecture and sensory integration, necessitate a cognitive adaptation phase. In cases of unilateral transplantation, complexities escalate, particularly in coordinating visual inputs between the transplanted and native eyes. Challenges such as ocular alignment, binocular fusion, and depth perception arise, where asynchronous visual signals can lead to diplopia or diminished visual clarity. The neural mechanisms underpinning these adaptive processes are still largely uncharacterized and represent an active area of ongoing research.

#### 4.1.2 Immune rejection

Immune rejection remains one of the most significant barriers to the success of WET. To mitigate rejection, current clinical and preclinical WET protocols employ immunosuppressive therapy, typically involving a triple-drug regimen comprising tacrolimus (a calcineurin inhibitor), mycophenolate mofetil (an antiproliferative agent), and prednisone (a corticosteroid). This combination has demonstrated efficacy in prolonging graft survival and reducing the incidence of acute rejection episodes (60). However, the effectiveness of these regimens varies among individuals due to genetic and immunological heterogeneity, complicating long-term management (61). Moreover, chronic use of immunosuppressants carries substantial risks, including increased susceptibility to opportunistic infections, nephrotoxicity, hepatotoxicity, metabolic disorders, and malignancies (62). Thus, maintaining a delicate balance between adequate immunosuppression and minimizing adverse effects is essential for successful WET outcomes.

Additional strategies under investigation include localized drug delivery systems, immune tolerance induction via donor-specific regulatory T cells, and immune-modulatory exosome therapy, all of which aim to reduce systemic immunosuppression requirements (63, 64). The use of human leukocyte antigen (HLA) matching and molecular profiling may also improve donor–recipient compatibility, thereby decreasing the likelihood of graft rejection. Despite these advances, long-term graft acceptance and immune stability remain critical hurdles. Future research should prioritize the development of targeted and less toxic immunomodulatory approaches explicitly tailored for WET transplantation.

#### 4.1.3 Blood supply and maintenance of intraocular pressure

Restoring retinal blood supply is challenging due to the complex ocular vascular anatomy and the presence of systemic diseases. Techniques such as microvascular nerve anastomosis and ophthalmic to superficial temporal (O-ST) vascular anastomosis show promise in addressing these challenges (65). However, fully restoring the blood supply and maintaining intraocular pressure remain unresolved issues. These factors are critical to the long-term survival of the transplant and the potential for visual recovery (6, 57).

#### 4.1.4 Surgical complexity

WET requires meticulous anastomosis of the ophthalmic vasculature and precise alignment of the donor optic nerve with the recipient’s central nervous system. The technical challenges necessitate specialized skills and advanced techniques. Although experimental models have demonstrated the feasibility of WET with partial functional integration, the translation of these techniques into routine clinical practice remains hindered by the extreme complexity of the procedure. Successful transplantation necessitates not only the restoration of ocular perfusion but also the potential for functional neuronal integration (57). Vascular anastomosis must be achieved without compromising perfusion pressure while simultaneously avoiding thrombotic complications or ischemia. Equally complex is the need to connect or regenerate the optic nerve, a central nervous system structure with limited intrinsic regenerative capacity. Continued innovation in microsurgical tools, real-time imaging guidance, neuroregenerative approaches, and surgical training will be necessary to improve procedural success rates and move WET closer to clinical application.

#### 4.1.5 Ethical issues

WET, while offering potential life-altering benefits for individuals with irreversible vision loss, presents complex ethical challenges. These include the inherent risks and surgical complexity, safeguarding donor autonomy and data, and ensuring the equitable allocation of scarce donor eyes (66). Transparent communication regarding the experimental nature, current limitations of medical knowledge, and uncertain long-term outcomes is crucial for patients and families to provide truly informed consent.

Patient autonomy is fundamentally challenged by the necessity for lifelong immunosuppression, which carries significant risks such as graft-versus-host disease, malignancies, organ dysfunction, and heightened infection susceptibility. Furthermore, privacy concerns arise, especially with combined whole-eye and facial transplants, due to the eye’s role in biometric identification and the potential for “identity transfer.” Multi-layered privacy protection mechanisms, including biometric data segregation, standardized image anonymization, and legal constraints, are therefore essential to mitigate these risks for both donors and recipients (67).

Given the severe global shortage of donor eyes, WET, as a high-cost experimental procedure, must be carefully considered against technically mature alternatives such as corneal transplantation. Establishing WET’s position through cost–benefit analyses, social equity assessments, and clinical necessity is vital. Prioritizing patients with severe ocular trauma who lack other treatment options, while balancing technological innovation with social welfare, is paramount to ensure ethical resource distribution and uphold the dignity of donors and recipients (68–70).

### 4.2 Strategies and research directions for overcoming challenges

#### 4.2.1 Application of stem cell technologies

Stem cell transplantation involves two main strategies: temporary and permanent implantation. Temporary implantation uses pluripotent stem or ocular progenitor cells in a non-polarized form, providing neuroprotective and immunomodulatory factors, with outcomes dependent on cell survival and secretory activity. Permanent implantation aims to replace damaged retinal cells with stem cell-derived photoreceptors or RPE cells, thereby restoring the atrophied cells of the same type (71, 72). The choice between these strategies depends on the extent of ocular damage, especially during WET. Both approaches can be evaluated at various stages of the transplant process and for different ocular tissues, which helps to ensure precise restoration of the missing tissue. Stem cell therapy also holds the potential to cure untreatable retinal degenerations that lead to blindness (73).

Stem cell-based approaches offer advantages such as reduced immunological rejection and tailored treatments for specific ocular diseases, making them promising for tissue repair and vision restoration after WET surgery. However, challenges persist, including the identification of reliable stem cell sources, the optimization of differentiation, the improvement of post-transplant survival, and the precise regeneration of ocular tissues. Continued research and innovative solutions are imperative to overcome these hurdles and fully realize the transformative potential of stem cell technologies in WET surgery.

#### 4.2.2 Advanced immunosuppressive protocols

Successful solid organ transplantation (SOT) demands precise immunosuppressive management, carefully balancing the prevention of graft rejection with the minimization of infection risk. While the “net state of immune suppression” concept aims to encompass all factors contributing to infection susceptibility, reliable assays for comprehensively evaluating immune function in immunosuppressed transplant recipients are still lacking, particularly in relation to infection control and allograft function. Quantitative viral load measurement offers a promising approach to assess integrated immune function and a recipient’s ability to manage latent viral infections (74).

Immunosuppressive therapies, while indispensable for preventing rejection, carry significant adverse effects, such as increased vulnerability to infections, malignancies, cardiovascular diseases, and nephrotoxicity. This highlights an urgent need for strategies that effectively mitigate these detrimental impacts. Extracorporeal photopheresis (ECP) has emerged as a promising adjunctive therapy, demonstrating its capacity to improve the management of acute and recurrent rejection, enhance allograft survival, and concurrently reduce the overall burden of immunosuppression, thereby addressing critical deficiencies in current therapeutic regimens (75).

Furthermore, achieving long-lasting, antigen-specific immune tolerance remains a formidable challenge across diverse medical domains, including autoimmunity, allergies, organ transplantation, and gene therapy. Recent breakthroughs in understanding the mechanisms of immune tolerance have opened up new avenues for research and present substantial therapeutic potential, directly addressing a pressing clinical need for more targeted and sustainable immune modulation strategies (76).

#### 4.2.3 Innovations in minimally invasive surgical techniques

Recent advancements in minimally invasive techniques show promise for treating complex ocular and facial conditions. One key innovation is laser chorioretinal anastomosis (L-CRA), which addresses central retinal vein occlusion (CRVO). L-CRA uses a high-power density laser to create an anastomosis between an obstructed retinal and choroidal vein, bypassing the blockage and restoring retinal venous outflow. When combined with intravitreal anti-VEGF agents, L-CRA may offer a permanent solution for CRVO, reducing the need for ongoing therapy and improving visual outcomes (77, 78).

The ophthalmic to superficial temporal artery (O-ST) bypass represents a significant advancement in facilitating the procurement of facial allografts that include the orbit. This technique enhances orbital and ocular perfusion by creating a bypass between the ophthalmic artery and the superficial temporal artery, which is a branch of the external carotid artery. Clinically, this approach enables successful facial allograft transplantation, complete with orbital components, by utilizing a single external carotid artery pedicle for primary perfusion, thereby substantially enhancing the feasibility of complex facial reconstruction involving the eye region (65).

These innovations underscore the increasing potential of minimally invasive techniques in treating complex ocular and facial conditions, yielding substantial improvements in both functional and esthetic outcomes.

#### 4.2.4 Three-dimensional (3D) printing and computer-assisted technologies

3D printing and advanced computer-assisted technologies have emerged as critical enablers in the field of WET, as evidenced by their significant contribution to the world’s first successful procedure (79). Specifically, the pioneering 3D technologies developed by the Belgian company Materialize played a crucial role in this groundbreaking surgery. These bespoke instruments, such as precision cutting guides and anatomical bone models, empowered surgeons to achieve faster and exceptionally accurate bone fragment positioning. Furthermore, 3D printing technology enables the customization of implant formats and sizes to precisely match the unique dimensions of each patient’s eye socket. The integration of computer-assisted technologies is dedicated to making vision-restoring WET a widespread reality. This sophisticated level of technological integration and customization not only elevated the precision and efficiency of the surgical intervention but also played a vital role in improving patient outcomes.

#### 4.2.5 Interdisciplinary collaborative research

**Figure 3:**
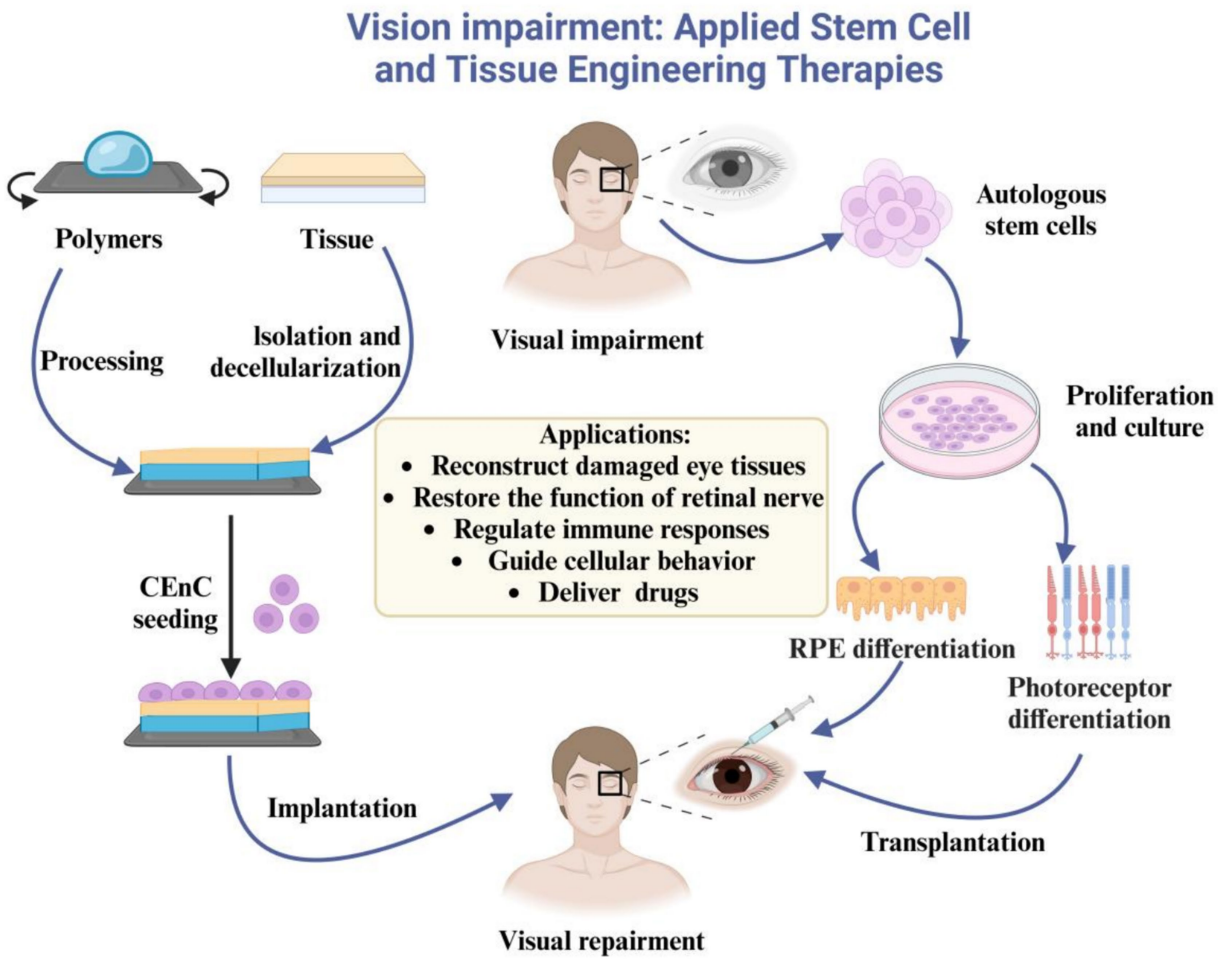
Application of stem cells and tissue engineering in the treatment of vision loss. Stem cell therapy involves the isolation and cultivation of autologous stem cells, followed by directed differentiation and reinfusion into the body. This approach not only reduces immunological rejection but also replenishes specific cells and repairs tissues. Tissue engineering utilizes biocompatible materials to provide a matrix for cell growth, induce cellular behaviors, and facilitate drug delivery.

Stem cells and tissue engineering represent pivotal interdisciplinary fields that hold substantial promise for addressing various severe ocular and facial injuries, including those that potentially require WET. Despite their therapeutic potential, significant challenges such as immune rejection and the complete restoration of visual function persist (Figure 3). To navigate these complexities, two primary stem cell-based strategies are actively explored: first, the temporary implantation of pluripotent stem cells or ocular progenitor cells, aimed at neuroprotection and immune modulation, and second, the permanent integration of stem cell-derived photoreceptors or retinal pigment epithelial (RPE) cells to replace damaged retinal tissues effectively. The selection of an appropriate strategy is critically dependent on the extent and specific nature of the tissue damage. While stem cell therapy offers a compelling solution for retinal degeneration by potentially reducing immune rejection and enabling personalized treatments, obstacles related to stem cell sourcing, survival rates post-transplantation, and effective tissue integration remain significant research foci (71, 72).

Complementing stem cell research, tissue engineering plays a crucial role in enhancing ocular tissue reconstruction by integrating biocompatible scaffolds with advanced cell culture and transplantation techniques (80). Maintaining corneal epithelial stability is crucial for preserving transparency and vision; however, conditions such as limbal stem cell deficiency can severely disrupt this critical balance. Historically, traditional transplant materials and allografts have frequently proven inadequate in providing satisfactory long-term outcomes for such conditions. Recent innovations include the development of decellularized human limbal scaffolds, which have demonstrated complete epithelialization and regeneration *ex vivo* when used for limbal epithelial progenitor cell transplantation (81). Furthermore, the design of suitable scaffolds is essential for facilitating tissue regeneration and enabling targeted cell and drug delivery in various ocular cell transplantations.

Collectively, these advancements in tissue engineering aim to overcome critical limitations such as donor shortages, facilitate robust tissue repair, and mitigate immune rejection. This offers considerable potential for significantly improved functional recovery across a spectrum of ocular conditions, including those associated with severe trauma or disease. However, the successful and widespread clinical translation of these sophisticated laboratory-based findings remains a substantial and ongoing challenge.

WET, combined with facial transplantation, represents a novel and evolving surgical strategy for managing severe ocular trauma and extensive facial injuries. Early clinical assessments of WET, specifically for traumatic ocular damage, have shown considerable promise, although the procedure remains highly experimental and necessitates rigorous investigation (5, 6). Despite these encouraging early outcomes, significant technical and immunological challenges persist. These include achieving successful vascular integration, promoting optic nerve regeneration, and ensuring long-term graft survival. Moreover, further systematic research and longitudinal studies are crucial for determining the long-term feasibility, safety, and functional outcomes of WET combined with facial transplantation (57).

In summary, this review highlights the recent breakthroughs in allogeneic WET, including the historic achievement of the world’s first successful transplant. The integration of advanced surgical techniques with cutting-edge stem cell biology demonstrates significant potential for visual recovery. Despite these advancements, critical challenges such as optic nerve regeneration and immune rejection persist, necessitating extensive research and innovation. Future progress in WET hinges on optimizing the optic nerve connection, refining immunosuppressive strategies, and leveraging advancements in regenerative medicine. Given the inherent complexity of this field, future efforts must prioritize interdisciplinary collaboration. This collaborative approach is essential to address current limitations and pave the way for WET to become a clinically viable solution for individuals suffering from severe visual impairments.
